# Acetate as an active metabolite of ethanol: studies of locomotion, loss of righting reflex, and anxiety in rodents

**DOI:** 10.3389/fnbeh.2013.00081

**Published:** 2013-07-10

**Authors:** Marta Pardo, Adrienne J. Betz, Noemí San Miguel, Laura López-Cruz, John D. Salamone, Mercè Correa

**Affiliations:** ^1^Àrea de Psicobiologia, Campus Riu Sec, Universitat Jaume ICastelló, Spain; ^2^Department of Psychology, Quinnipiac UniversityHamden, CT, USA; ^3^Department of Psychology, University of ConnecticutStorrs, CT, USA

**Keywords:** ataxia, anxiety, alcohol metabolism, acetaldehyde, acetate, narcosis

## Abstract

It has been postulated that a number of the central effects of ethanol are mediated via ethanol metabolites: acetaldehyde and acetate. Ethanol is known to produce a large variety of behavioral actions such anxiolysis, narcosis, and modulation of locomotion. Acetaldehyde contributes to some of those effects although the contribution of acetate is less known. In the present studies, rats and mice were used to assess the acute and chronic effects of acetate after central or peripheral administration. Male Sprague-Dawley rats were used for the comparison between central (intraventricular, ICV) and peripheral (intraperitoneal, IP) administration of acute doses of acetate on locomotion. CD1 male mice were used to study acute IP effects of acetate on locomotion, and also the effects of chronic oral consumption of acetate (0, 500, or 1000 mg/l, during 7, 15, 30, or 60 days) on ethanol- (1.0, 2.0, 4.0, or 4.5 g/kg, IP) induced locomotion, anxiolysis, and loss of righting reflex (LORR). In rats, ICV acetate (0.7–2.8 μmoles) reduced spontaneous locomotion at doses that, in the case of ethanol and acetaldehyde, had previously been shown to stimulate locomotion. Peripheral acute administration of acetate also suppressed locomotion in rats (25–100 mg/kg), but not in mice. In addition, although chronic administration of acetate during 15 days did not have an effect on spontaneous locomotion in an open field, it blocked ethanol-induced locomotion. However, ethanol-induced anxiolysis was not affected by chronic administration of acetate. Chronic consumption of acetate (up to 60 days) did not have an effect on latency to, or duration of LORR induced by ethanol, but significantly increased the number of mice that did not achieve LORR. The present work provides new evidence supporting the hypothesis that acetate should be considered a centrally-active metabolite of ethanol that contributes to some behavioral effects of this alcohol, such as motor suppression.

## Introduction

Acetate is a short-chain fatty acid formed as the final step in ethanol oxidation. The oxidative metabolism of ethanol into acetaldehyde takes place in several organs, and can involve multiple enzymes, including alcohol dehydrogenase (ADH), cytochrome P450 2E1 (CYP2E1), and catalase. Acetaldehyde is then metabolized mainly by aldehyde dehydrogenase (ALDH) into acetate. Following ethanol ingestion, approximately 70% of the acetate generated through oxidative metabolism is released from the liver into systemic circulation (Busch, 1953; Van den Berg et al., 1966). Acetate can be detected in plasma after ethanol administration, because the portion that has not been metabolized hepatically is released into the blood. Acetate is then redistributed throughout the body, metabolized in extra-hepatic organs (Lundquist et al., 1962), rapidly taken up into the brain by a carrier-mediated process (Oldendorf, 1973), and also is actively metabolized in the brain (Cullen and Carlen, 1992). An alternative central source of acetate is brain ethanol metabolism. It has been demonstrated (Zimatkin et al., 2006) that pharmacological manipulations that reduce catalase activity also reduce the amount of acetate detected in rat and mice brain homogenates. Moreover, when brain homogenates from CYP2E1 KO mice where incubated with ethanol plus a catalase inhibitor, there was a significant reduction of acetate formation, an effect which was not observed in brain homogenates from catalase-deficient mice (Zimatkin et al., 2006). Pharmacological inhibition of CYP2E1 also leads to significant decreases in acetate accumulation in rat brain homogenates. Moreover, enzymatic inhibition of ADH and ALDH also reduced acetate levels (Zimatkin et al., 2006). These results demonstrate that acetate can be formed in the brain via ethanol metabolism and that the enzymatic systems involved in this process are some of the ones required to form acetaldehyde.

Acetate has been demonstrated to have specific effects on behavior. Peripherally administered acetate increased the time off a treadmill, a measure of motor incoordination in rats, and suppressed locomotion in mice (Carmichael et al., 1991; Israel et al., 1994). In fact, peripherally injected acetate has been demonstrated to be three times more potent than ethanol at suppressing locomotion in mice (Israel et al., 1994). Moreover, acetate injected peripherally or in the brain ventricles also suppressed food-reinforced lever pressing on a FR5 schedule of reinforcement, which generates high levels of performance (Arizzi et al., 2003; McLaughlin et al., 2008). Thus, it has been suggested that acetate is involved mainly in the depressant effects of ethanol (Carmichael et al., 1991; Israel et al., 1994; Arizzi et al., 2003; Correa et al., 2003). Consistent with this idea, acetate can mimic some of the motor suppressant, ataxic, or sedative effects of ethanol. For instance, general anesthesia is potentiated in a dose-dependent fashion by ethanol as well as acetate (Carmichael et al., 1991; Campisi et al., 1997). Acetate seems to mediate tolerance to the loss of the righting reflex (LORR) produced by ethanol. Repeated administration of ethanol [3.5 g/kg, intraperitoneal (IP) during 7 days] to outbred rats, resulted in tolerance to LORR induced by ethanol and to higher concentrations of acetate in different areas of the brain compared to acutely treated animals (Kiselevski et al., 2003). Moreover, higher amounts of acetate are formed in short sleeping (SS) rats, which have an inborn tolerance to the LORR induced by high doses of ethanol, relative to the long sleeping (LS) substrain (Zimatkin et al., 2011).

Because direct administration of ethanol and acetaldehyde seem to have different motor effects depending on the route of administration (for a review see Correa et al., 2012), the present experiments addressed the potential differences between peripheral and central injections of acetate on locomotor activity in rats. We also evaluated the impact of peripherally administered acetate on motor activity in mice at similar low doses. In a second group of experiments, because acetate accumulation after repeated administration of ethanol seems to mediate tolerance to LORR induced by ethanol (Kiselevski et al., 2003), we evaluated the impact of chronic consumption of acetate across multiple time periods on different behaviors modulated by an acute dose of ethanol in mice. Thus, we evaluated the impact of chronic exposure to acetate on ethanol-induced stimulation of locomotion and on ethanol-induced LORR in mice. Moreover, although acutely administered acetate has not shown to have an effect on anxiety measures in mice (Escrig et al., 2007, 2012) and rats (Correa et al., 2003), the anxiolytic actions of ethanol at low doses are well known (Correa et al., 2008). Thus, in the present study we also evaluated the impact of chronic administration of acetate on measures of anxiolysis induced by a low dose of ethanol in mice.

## Methods

### Subjects

Male Sprague-Dawley rats (Harlan Sprague-Dawley, Indianapolis, IN), were housed in a colony maintained at 23°C with lights on from 7:00 to 19:00 h. Animals weighed between 350 and 430 g at the time of the experiment. These animals had *ad libitum* access to food and water in their home cages. Before the test day, rats were allowed 2 weeks to acclimate to laboratory conditions, plus 1 week of being handled by the experimenter for 5 min each day. For the IP study, a total of 43 rats (*n* = 8–9 per group) were used and for the intraventricular (ICV) study the number was 38 (*n* = 8–10 per group).

CD1 male mice (30–40 g) were purchased from Harlan-Interfauna Iberica S.A. (Barcelona, Spain). Mice 6–7 weeks old at the beginning of experiments were housed in groups of three per cage, with standard laboratory rodent chow and tap water available *ad libitum*. They were maintained in the colony at 22 ± 1°C with lights on from 8:00 to 20:00 h. Mice were handled and habituated to the test room for 1 week before tests were conducted. For the acute acetate study, 42 mice were used (*n* = 10–11 per group). For the chronic acetate studies, the locomotion experiment included 78 mice (*n* = 8–9 per group), the anxiety experiment included a total of 63 mice (*n* = 10–11 per group), and for the LORR experiments the total number was 218 (*n* = 14 per group).

All experimental procedures were approved by the Institutional Animal Care and Use Committee, and complied with the European Community Council directive (86/609/ECC) for the use of laboratory animal subjects and with the “Guidelines for the Care and Use of Mammals in Neuroscience and Behavioral Research” (National Research Council, 2003).

### Drugs and selection of doses

Anhydrous sodium acetate (hereafter referred to as acetate, Fisher Scientific) was dissolved in physiological saline for the IP studies, in artificial cerebrospinal fluid (aCSF) for the ICV studies, and in tap water for the oral chronic studies. These vehicles serve as the control solutions. For IP injections, acetate 10% w/v was used as the stock solution from which the different doses were obtained. ICV acetate doses of 0.7, 1.4, or 2.8 μmoles (0.0, 42.03, 84.07, or 168.14 μg), were administered in 1.0 μl total volume. Chronically administered acetate was prepared dissolving sodium acetate in tap water. Concentration of the solutions were 500 or 1000 mg/l. After recording fluid intake and body weight per animal for 60 days, we calculated that the average dose of acetate consumed for the group exposed to 500 mg/l was 29.9 ± 5.3 mg/kg and for the 1000 mg/l group was 67.6 ± 1.8 mg/kg. Ethanol (96% v/v, Panreac Quimica S. A.) was dissolved in physiological saline in a 20% v/v solution used as the stock solution from which the different doses were obtained. Hydrochloric acid (1N, Panreac Quimica S. A.) was used to bring the sodium acetate solutions for the acute studies to pH 7.4. Xylazine and Ketamine were purchased from Phoenix Pharmaceutical, Inc. (St. Joseph, Mo).

The selection of doses and times was based on pilot studies and on previous studies from our laboratory (Arizzi et al., 2003; Arizzi-LaFrance et al., 2006; Correa et al., 2003; Escrig et al., 2012).

### Surgical procedure and ICV injections

For the ICV study, rats were implanted with unilateral guide cannulae (10.0 mm length, 23 ga.). Rats were anesthetized with a solution (1.0 ml/kg, IP) that contained Ketamine (100 mg/ml) and Xylazine (20 mg/ml). The stereotaxic coordinates for the cannulation into the lateral ventricle were as follows: AP −0.5 mm (from bregma), DL +1.3 mm lateral (from midline), and DV −3.0 mm ventral (from the surface of the skull). The incisor bar on the stereotax was set to 0.0 mm above the interaural line. All animals were single housed following surgery, and were allowed to recover for 7–10 days before behavioral testing. Stainless steel stylets were kept in each guide cannulae to maintain its integrity.

ICV injections were made via 30 ga. stainless steel injection cannulae extending 1.5 mm below the guide cannulae. The injectors were attached to 10.0 μl Hamilton syringes by PE-10 tubing, and were driven by a syringe pump (Harvard Apparatus) at a rate of 0.5 μl/min for a total volume of 1.0 μl. Following the infusion period the injectors were left in place for 1 min to allow for diffusion of the drug, after which the injectors were removed, stylets were replaced, and animals were immediately placed into the behavioral chambers for testing.

### Histology

For the ICV experiments, the placements of the injectors were verified histologically. After the experiments were completed, all animals were intracardially perfused with heparinized physiological saline. Brains were stored refrigerated in 3.7% formaldehyde solution for at least 5 days prior to slicing. Consecutive 50 micron sections through the relevant brain areas were collected, mounted on slides, and stained with cresyl violet solution to aid in detection of the injector tracts. Coverslipped slides were viewed microscopically to assess accuracy of implantation. Any animal with improper placement, or significant damage around the injection site, was not included in the statistical analyses of behavioral data. A total of 5 animals were rejected due to bad placements.

### Apparatus and behavioral procedure in rats

#### Enclosed stabilimeter

Locomotor testing was performed in an automated activity chamber (28 × 28 × 28 cm), which was inside a sound-proof shell. The floor of the chamber consisted of two moveable wire mesh panels (27 × 13 cm) mounted 6.0 cm above the chamber floor on a center rod attached at either end to the sides of the chamber; this allowed for slight vertical movement of the floor panels. Movement of the panels was detected by microswitches mounted outside the chamber at the ends of the panels. A depression of a given quadrant (quadrant = 1/2 of each panel) would close the circuit on the microswitch attached to the panel. Each microswitch closure was counted as a single activity count, and activity counts were recorded by a computer in 10 min intervals. Rats were habituated to the chamber and to injections prior to the drug test. This was done to decrease activational effects due to novelty on the test day. On the test day, animals were placed into the activity chamber immediately after IP injections, and for the ICV studies they were placed in the chambers after 1 min to allow for diffusion of the drug, as described above. Locomotion was recorded in 10 min periods. In the ICV studies, after drug injections animals were anesthetized and perfused as described above, and histological analyses of brain sections were performed.

### Apparatus and behavioral procedures in mice

#### Enclosed activity box

The enclosed locomotion chamber was made of polypropylene and consisted of a square white box divided in two compartments (25 cm *W* × 25 cm *H* × 22 cm *L*), covered with a translucent ceiling. The behavioral test room was illuminated with a soft red light, and external noise was attenuated. As in the stabilimeter, this enclosed two-compartment box was used in order to minimize anxiogenic stimulation of locomotion. Mice were habituated to the chamber and to injections prior to the drug test. This was done to decrease activational effects due to novelty on the test day. Acetate IP was injected 10 min before test started. Locomotion was recorded for 10 min. An activity count was registered by a trained observer, unaware of the experimental condition, each time the animal crossed from one quadrant to another with all four legs.

#### Open field (OF)

The OF arena consisted of a Plexiglass cylinder with translucent walls (30 cm in diameter and 30 cm high) and an opaque floor divided into four equal quadrants by two intersecting lines. Mice were handled repeatedly and habituated to the test room before the behavioral test, but were not pre-exposed to the OF. On the test day, ethanol (1.0 or 2.0 g/kg) or saline were administered acutely IP and animals were placed immediately in the OF and locomotor observations started 10 min later. The behavioral test room was illuminated with a soft light, and external noise was attenuated. An activity count was registered by a trained observer, unaware of the experimental condition, each time the animal crossed from one quadrant to another with all four legs.

#### Dark-light box

The apparatus consisted of a polypropylene chamber divided in two compartments by a partition containing a small opening (5 cm *H* × 5 cm *W*). The light compartment (25 cm *W* × 25 cm *H* × 25 cm *L*) was open, painted in white, and illuminated, while the dark compartment (25 cm *W* × 25 cm *H* × 18 cm *L*) was painted in black and enclosed by a removable ceiling. This anxiety paradigm measures the avoidance that rodents show to bright open spaces. Several parameters were recorded during 5 min testing sessions. The dependent variables were: latency for the first entry into the bright compartment from the dark one, latency to go back to the dark compartment, total time spent in the bright compartment, and total crosses between compartments. In the acute study, acetate IP was injected 10 min before the dark-light box test.

#### LORR

Test of latency and duration of LORR were recorded consecutively. Ethanol (4.0 or 4.5 g/kg) was injected IP, and immediately mice were individually placed in a plexiglass cage. The latency was defined as the time elapsed between ethanol injection and LORR. Mice that did not lose righting reflex were not included in the posterior measurements. After mice lost the righting reflex, they were put on their back in a V-shape bed. The duration of LORR was defined as the time elapsed from LORR to the time that righting reflex was regained. Recovery was determined when mice could right themselves twice in 1 min after being placed on their backs. All the animals recovered the righting reflex. The behavioral room was illuminated with a soft light and external noise was attenuated.

These parameters were chosen based on previous studies (Correa et al., 1999, 2001, 2003, 2008; Arizzi-LaFrance et al., 2006; Chuck et al., 2006; Escrig et al., 2012).

### Statistical analysis

All the experiments used a between-groups design, with each animal only being tested once. Data were analyzed by simple analysis of variance (ANOVA). If there was a significant overall drug effect, the LSD was used to make planned comparisons between each dose and the respective vehicle control condition. A computerized statistical program was used to analyze these data (SPSS 10.0).

## Results

### Experiment 1: effect of acute central or peripheral administration of acetate on locomotor activity in rats

**Figure 1:**
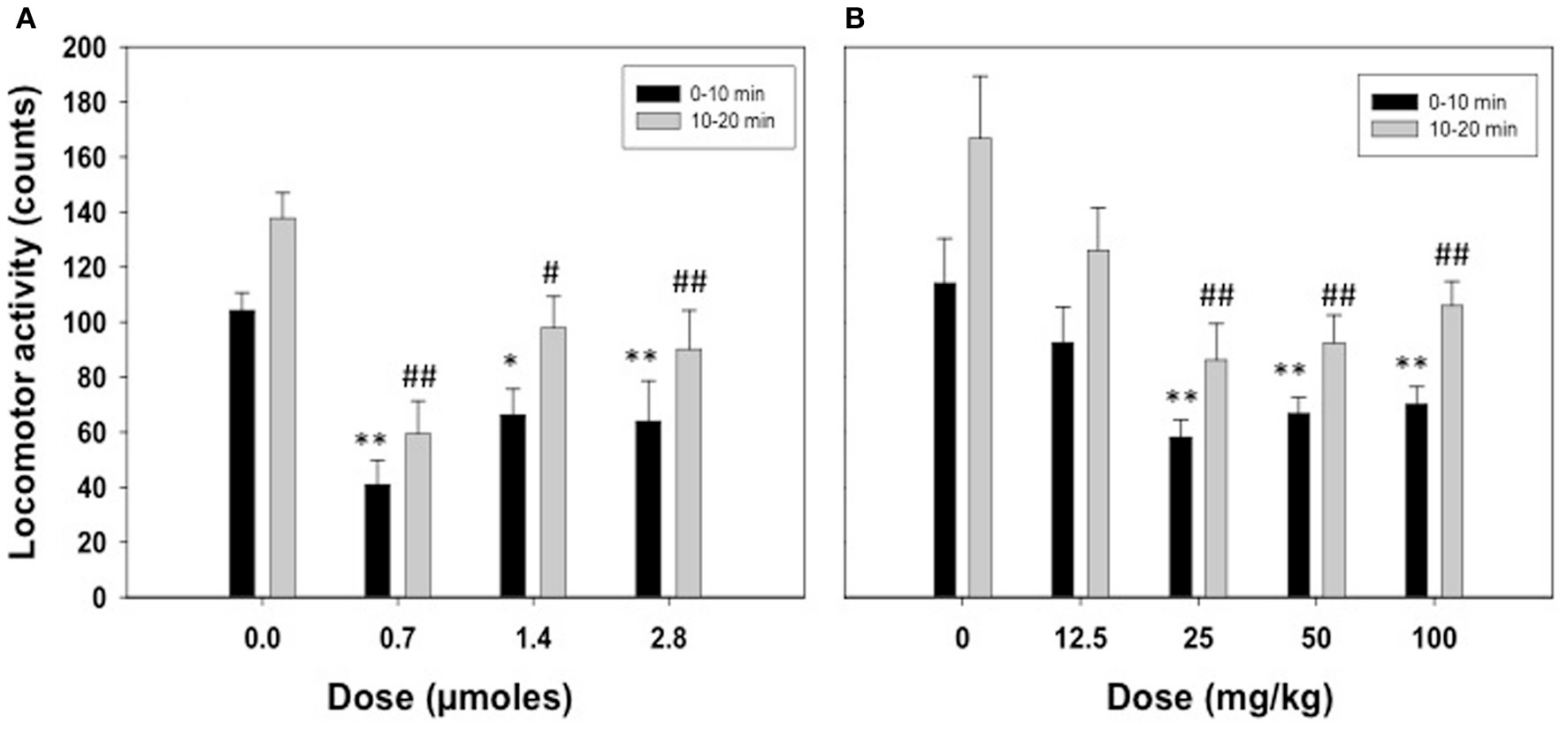
**Effect of acetate administered acutely ICV (A) or IP (B) on locomotor activity evaluated in rats in the enclosed stabilimeter**. Data are expressed as mean + SEM counts in 10 min periods. ^**^p < 0.01, ^*^p < 0.05 different from vehicle in the 0–10 min period. ^##^*p* < 0.01, ^#^*p* < 0.05 different from vehicle in the 10–20 min period.

Figure 1A shows the effect of ICV acetate administration (0.0, 0.7, 1.4, or 2.8 μmoles) on locomotor activity in the stabilimeter. Because the pattern of results was the same in the two time periods registered and there was no interaction, separate ANOVAs were performed for the two periods. The One-Way ANOVA for the 0–10 period showed a statistically significant overall treatment effect [*F*_(3, 22)_ = 7.82, *p* < 0.01]. Planned comparisons showed all doses of acetate were significantly different from vehicle (0.7 and 2.8 μmoles *p* < 0.01, and 1.4 μmoles *p* < 0.05). The same pattern of results were found for the ANOVA of the second period [*F*_(3, 22)_ = 8.47, *p* < 0.01]. The data for the effect of IP acetate administration (0, 12.5, 25, 50, or 100 mg/kg) on locomotor activity in the stabilimeter were analyzed in the same way (see Figure 1B). The One-Way ANOVA for the first period of time showed a significant effect of the peripheral dose of acetate [*F*_(4, 36)_ = 4.90, *p* < 0.01], and the planned comparisons showed that the three highest doses were significantly different from vehicle (*p* < 0.01). The same results were shown for the second period of time; 10–20 min [*F*_(4, 36)_ = 4.86, *p* < 0.01], and for the planned comparisons.

### Experiment 2: effect of acute IP administration of acetate on locomotor activity in mice

**Figure 2:**
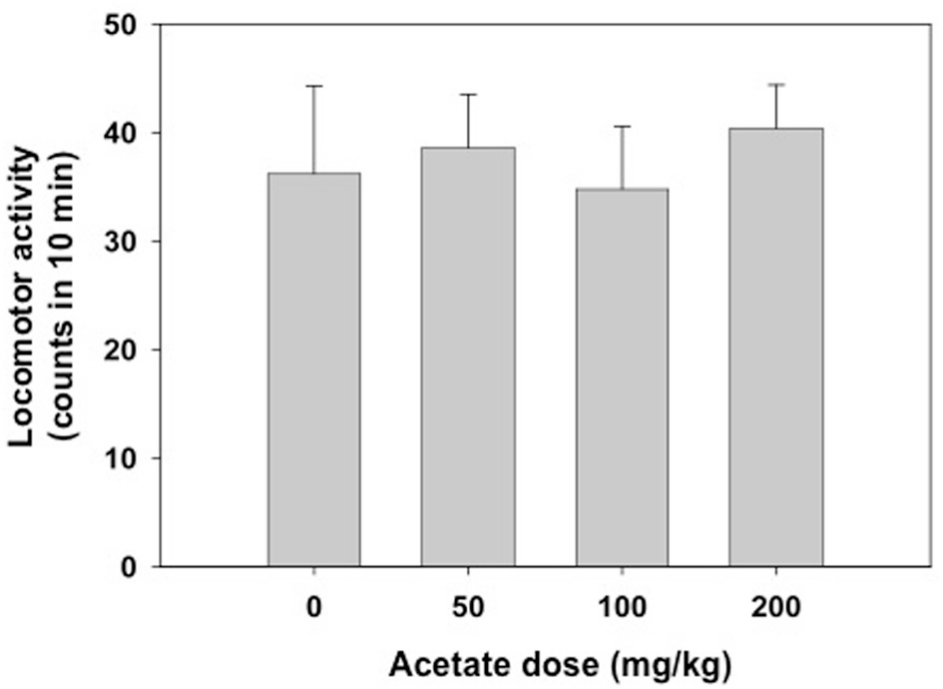
**Effect of acetate administered acutely IP on locomotor activity evaluated in mice in the enclosed activity box**. Data are expressed as mean + SEM counts in 10 min.

The one-way factorial ANOVA for the effect of acetate treatment (0, 50, 100, or 200 mg/kg) did not show significant effects on the number of crossings between the two compartments of the enclosed box [*F*_(3, 38)_ = 0.63, n.s.]. These data are shown in Figure 2.

### Experiment 3: effect of 15 days of oral consumption of acetate on ethanol-induced locomotion in mice

**Figure 3:**
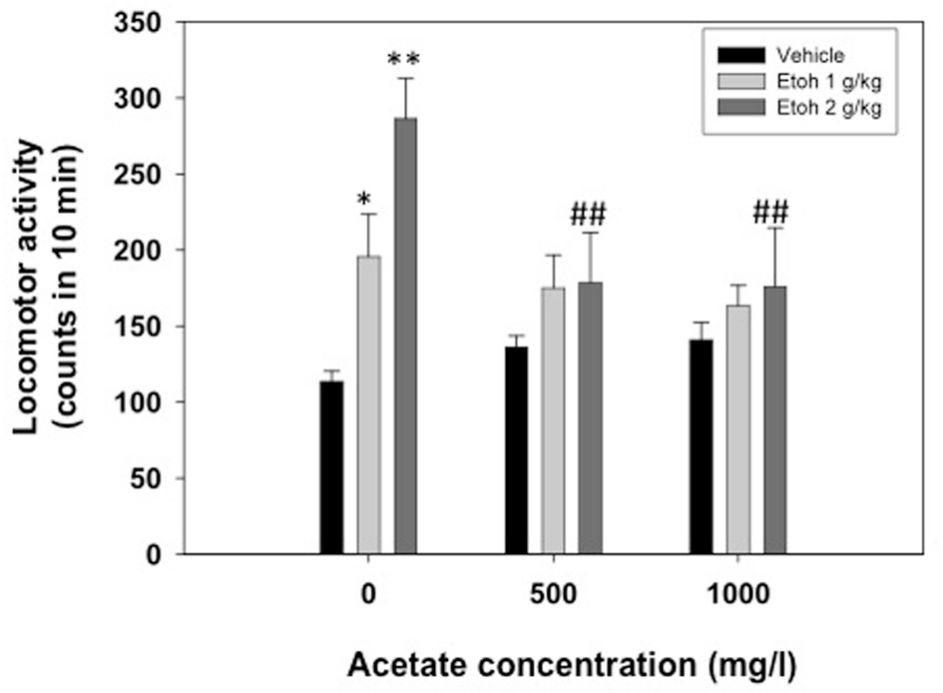
**Effect of chronic acetate consumption during 15 days on ethanol-induced locomotor activity in an open field in mice**. Data are expressed as mean ± SEM counts in 10 min. ^**^p < 0.01, ^*^p < 0.05 different from vehicle in the same acetate group. ^##^*p* < 0.01 different from the same dose of ethanol in the 0 mg/l group.

A two-way factorial ANOVA (concentration of acetate × dose of ethanol) showed no effect of the acetate concentration factor [*F*_(2, 69)_ = 1.42, n.s.], but a significant effect of the ethanol dose factor [*F*_(2, 69)_ = 9.50, *p* < 0.01], and a significant interaction [*F*_(4, 69)_ = 2.76, *p* < 0.05]. Planned comparison revealed that the two doses of ethanol significantly induced locomotion (1.0 g/kg *p* < 0.05 and 2.0 g/kg *p* < 0.01) compared to vehicle in the water-consuming group. Moreover, these differences disappeared in the acetate consuming groups. These results are depicted in Figure 3.

### Experiment 4: effect of 15 days of oral consumption of acetate on ethanol-induced anxiolysis in mice

The four dependent variables (see Table 1) were analyzed independently. A two-way factorial ANOVA (concentration of acetate × dose of ethanol) was performed in every case. The results of the ANOVA for the dependent variable latency to enter the bright compartment showed that there was a significant effect of the ethanol dose [*F*_(1, 57)_ = 4.72, *p* < 0.05], but no effect of the acetate treatment [*F*_(2, 57)_ = 0.36, n.s.], and no significant interaction [*F*_(2, 57)_ = 0.57, n.s.]. The same pattern of results for the dependent variable latency to come back to the dark compartment was found: ethanol dose [*F*_(1, 57)_ = 5.32, *p* < 0.05], the concentration of acetate [*F*_(2, 57)_ = 0.46, n.s.], and the interaction [*F*_(2, 57)_ = 0.78, n.s.]. These results demonstrate that ethanol had an anxiolytic effect independently of the acetate treatment. The results for the total time in the bright compartment showed no significant effect: ethanol dose [*F*_(1, 57)_ = 2.67, n.s.], acetate treatment [*F*_(2, 57)_ = 0.82, n.s.], and the interaction [*F*_(2, 57)_ = 0.68, n.s.]. The frequency of crossings between the bright and the dark compartments showed a marginally non-significant effect of the ethanol factor [*F*_(1, 57)_ = 3.28, *p* = 0.07], a significant effect of the acetate treatment [*F*_(2, 57)_ = 3.53, *p* < 0.05], but no significant interaction [F_(2, 57)_ = 0.52, n.s.].

**Table 1:** **Effect of chronic acetate consumption during 15 days on vehicle or ethanol (1 g/kg, IP) treated mice in measures of anxiety in the dark/light box**.

**Acetate (mg/l)**	**Latency to lit compartment**		**Latency to go back to dark compartment**		**Time in lit compartment**		**Number of crossings into the lit compartment**	
	**Veh**	**EtOH**	**Veh**	**EtOH**	**Veh**	**EtOH**	**Veh**	**EtOH**
0	11.6 ± 1.8	9.4 ± 1.6	6.6 ± 1.9	14.6 ± 2.9	105.9 ± 10.9	122.8 ± 14.5	27.7 ± 3.1	30.8 ± 3.7
500	11.2 ± 1.9	6.6 ± 1.5	7.5 ± 1.8	9.6 ± 1.4	128.2 ± 10.6	129.3 ± 6.6	27.2 ± 3.3	37.4 ± 2.8
1000	15.3 ± 6.5	7.2 ± 1.3	8.7 ± 1.4	12.4 ± 3.2	110.0 ± 11.2	137.1 ± 11.6	37.2 ± 4.8	40.8 ± 4.1

Data are expressed as the mean ± SEM seconds or counts in 5 min.

### Experiment 5: effect of oral consumption of acetate during different periods of time on ethanol-induced LORR in mice

**Figure 4:**
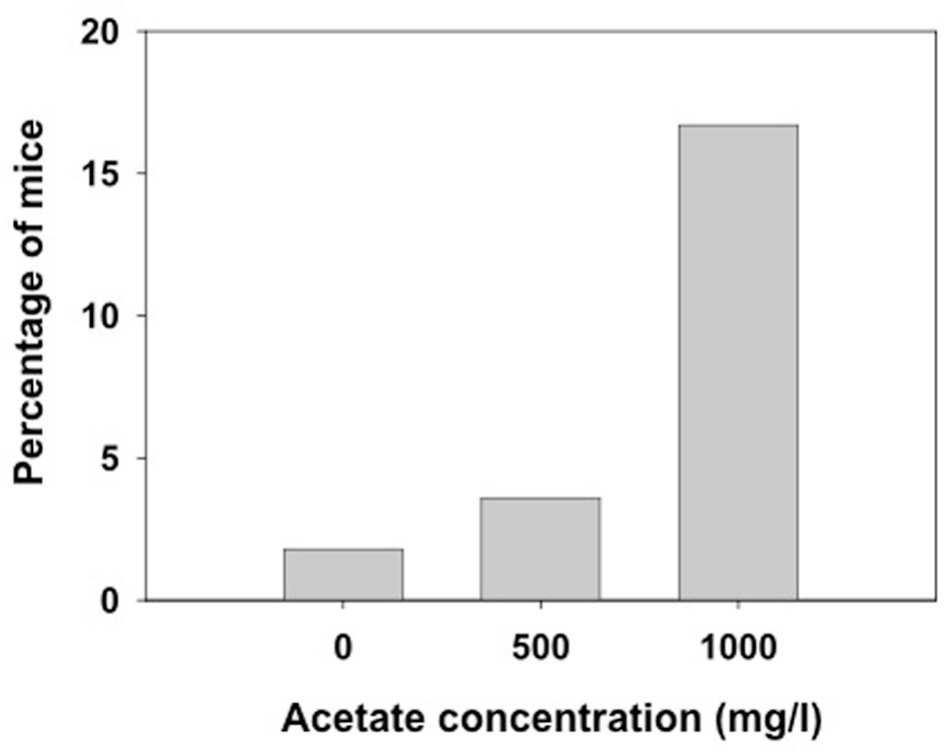
**Percentage of mice exposed to different concentrations of acetate that did not achieve LORR after receiving 4.0 g/kg ethanol IP**.

Animals received ethanol only once and different measures were assessed. We observed that the lower dose of ethanol (4.0 g/kg) did not produce LORR in some animals that were immediately excluded from the following measures in this experiment (they are not included in the latency and duration analyses). Grouping together the number of animals in the three treatment groups (water, 500 and 1000 mg/l) independently of how many days they had consumed acetate (15, 30, or 60 days, there were no animals in the 7 days groups), the χ^2^ test for independence showed a significant effect of the acetate treatment (χ^2^ = 10.64, *df* = 2, *p* < 0.01). These data are depicted as percentage of animals not achieving LORR in every treatment group in Figure 4.

**Figure 5:**
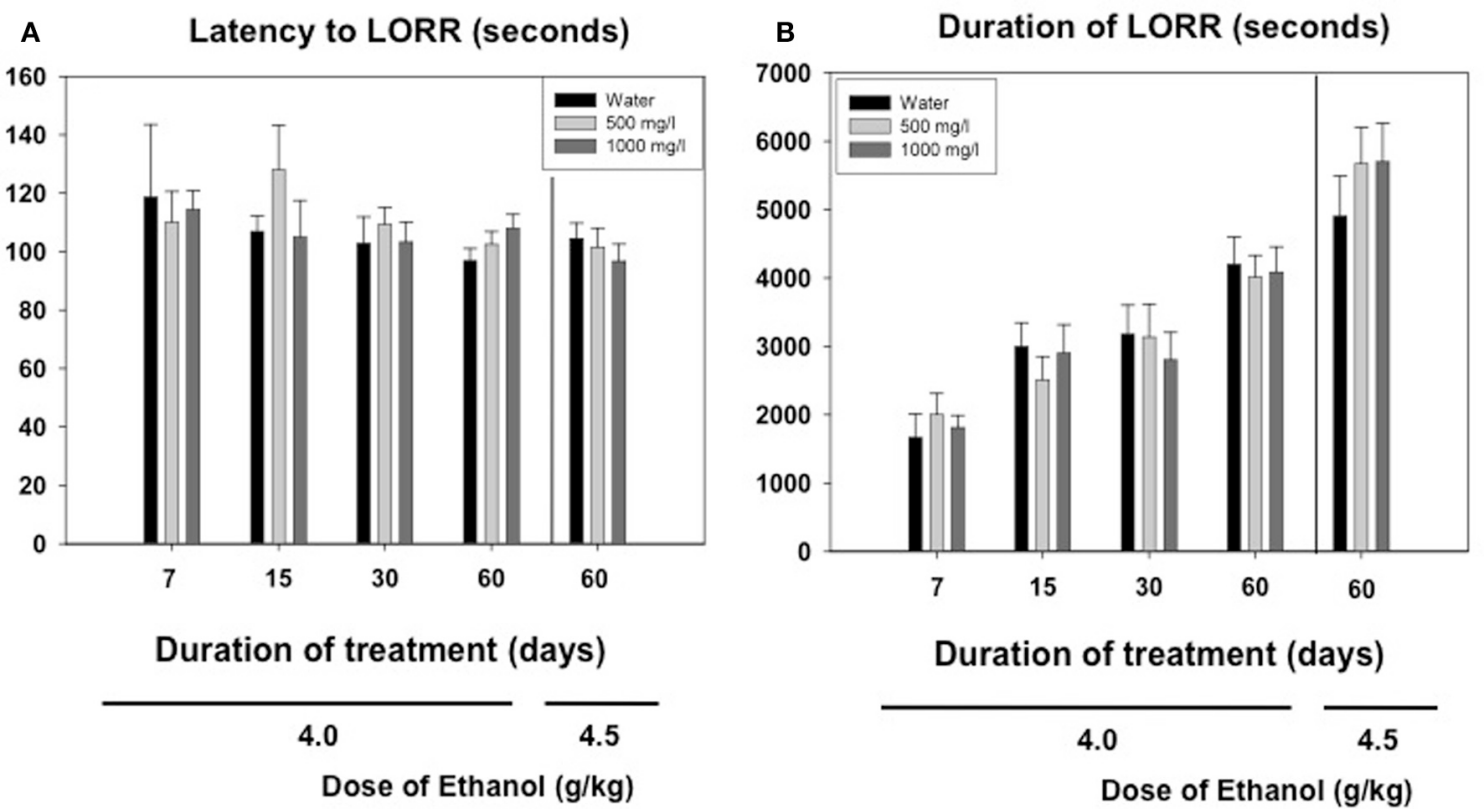
**Effect of chronic acetate consumption during different periods of time on latency (A) and duration (B) to LORR induced by an acute administration of ethanol (4.0 or 4.5 g/kg, IP)**. Data are expressed as mean ± SEM of time in seconds.

Among the animals that did achieve LORR, a two-way factorial ANOVA (concentration of acetate × time of consumption) for the latency to reach LORR measure yielded no significant effect of acetate concentration [*F*_(2, 163)_ = 0.54, n.s.], no effect of time of consumption [*F*_(3, 163)_ = 1.48, n.s], and no interaction [*F*_(6, 163)_ = 0.79, n.s.]. The factorial ANOVA for duration of LORR demonstrate no effect of the acetate concentration [*F*_(2, 163)_ = 0.07, n.s.], but a significant effect of the time of consumption [*F*_(3, 163)_ = 14.28, *p* < 0.01]. However, the interaction was not significant [*F*_(6, 163)_ = 0.20, n.s.]. Thus, 4.0 g/kg ethanol produced an increase in duration of LORR in older animals independently of the acetate treatment. The data for the higher dose of ethanol (4.5 g/kg) in animals consuming acetate during 60 days were analyzed separately by means of a One-Way ANOVA. The results show no effect of the concentration on either the latency [*F*_(2, 40)_ = 0.43, n.s.], or the duration of LORR [*F*_(2, 40)_ = 0.62, n.s.]. These data are presented in Figures 5A,B.

### Experiment 6: effect of oral consumption of acetate during different periods of time on volume of water consumed and body weight gain

**Figure 6:**
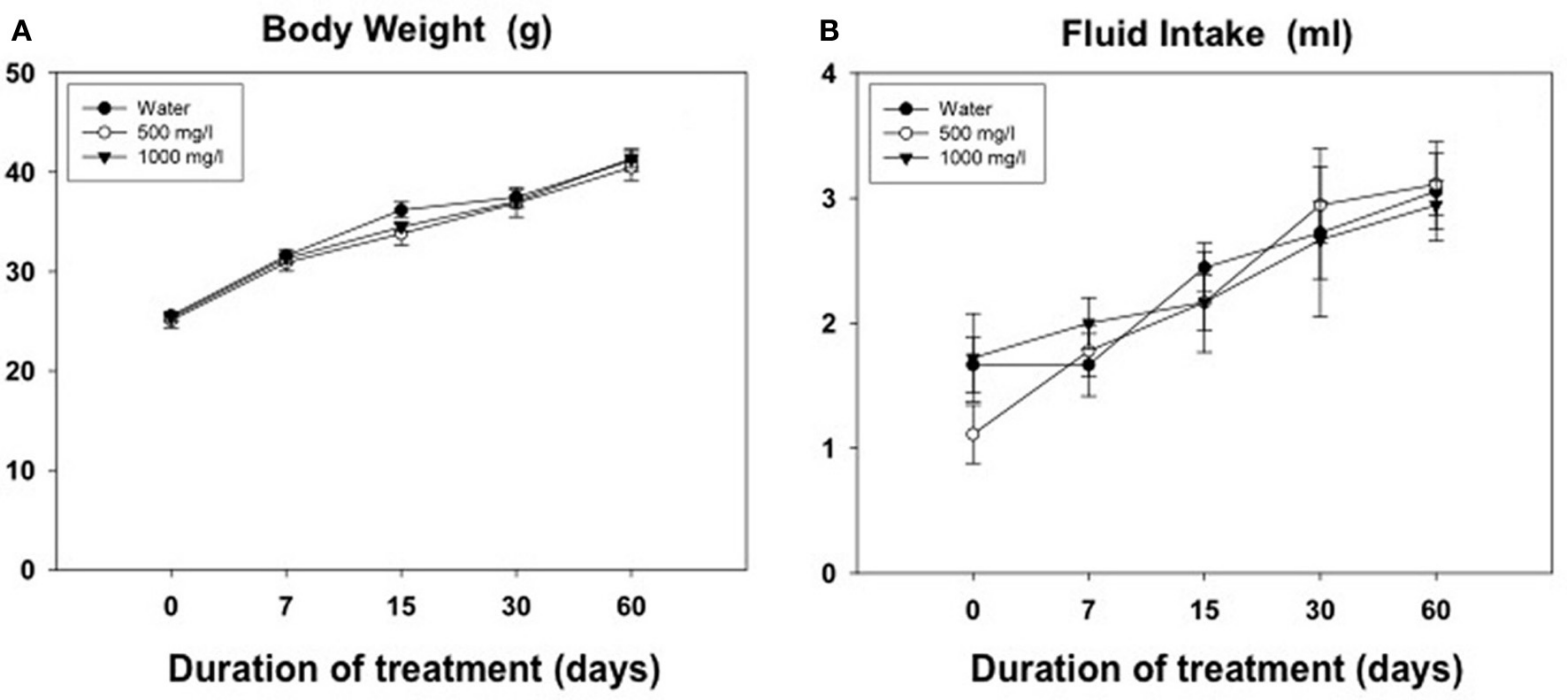
**Evolution of body weight (g) (A) and volume of fluid consumed (ml) (B) in animals exposed for 60 days to different concentrations of acetate**. Mean ± SEM of grams.

Results from the evolution of body weight and fluid intake in animals for experiment 5 are shown in Figures 6A,B. The Two-Way ANOVA for the body weight was analyzed with a within factor for duration of treatment and a between factor for concentration of acetate. There was a significant effect of the duration [*F*_(4, 960)_ = 437.5, *p* < 0.01], but no effect of concentration [*F*_(2, 10)_ = 0.40, n.s.], and no significant interaction [*F*_(8, 2)_ = 0.84, n.s.]. The same pattern of results was shown for the fluid intake variable. The Two-Way ANOVA showed a significant effect of the duration [*F*_(4, 11)_ = 15.43, *p* < 0.01], but no effect of concentration [*F*_(2, 10)_ = 0.11, n.s.], and no significant interaction [*F*_(8, 1)_ = 0.58, n.s.].

## Discussion

Studies of the behavioral effects of the ethanol metabolite acetaldehyde have been increasing in number, especially during the last decade; as a result, our knowledge of acetaldehyde's behavioral and neurochemical effects is quite comprehensive (for a recent review see Correa et al., 2012). However, acetate has remained mostly unknown, and only a handful of studies have addressed its behavioral and neurochemical actions (Israel et al., 1994; Correa et al., 2003; Kiselevski et al., 2003; Arizzi-LaFrance et al., 2004; McLaughlin et al., 2008; Zimatkin et al., 2011; Escrig et al., 2012). The present results demonstrate that acute low doses of acetate administered peripherally or into the ventricles reduce spontaneous locomotion in rats at least during 20 min (see Figures 1A,B). The present studies measured locomotion in small and enclosed stabilimeter cages. Centrally administered acetate (ICV) has also been shown to produce locomotor suppressant effects in rats in an open field arena (Correa et al., 2003). In that case acetate produced a monotonic decrease in activity (1.4 and 2.8 μmoles) marked by significant decreases in locomotion as well as rearing (Correa et al., 2003). The suppression is more efficacious when using the small stabilimeter cages (0.7 μmoles also suppressed locomotion), possibly because this device is less anxiogenic than the open field, and therefore induces a higher level of locomotion.

Rats seem to be more sensitive than mice to the suppressant effects of peripherally administered ethanol and acetate. Thus, in the present studies acetate doses between 25 and 100 mg/kg reduced locomotion in rats but not in mice; even the dose of 2.0 mg/kg did not suppress locomotion in mice under the present conditions (enclosed activity box); this dose is much lower than doses used in previous studies in mice (Israel et al., 1994). In mice the minimal dose of acetate effective for suppressing locomotion in an open field was 1.0 g/kg, while the dose of ethanol was 3.0 g/kg (Israel et al., 1994). Thus, acetate seems more potent than ethanol at suppressing locomotion. This difference in drug potency has also been observed in other studies in rats. When injected peripherally, acetate was more potent than ethanol or acetaldehyde for suppressing food-reinforced operant responding (Arizzi et al., 2003; McLaughlin et al., 2008), reducing the number of fast responses and increasing the number of pauses that the animals took during the operant session at doses of 200–400 mg/kg, IP (McLaughlin et al., 2008). Injected into the ventricles, acetate suppressed lever pressing (2.8 and 5.6 μmoles), and also increased the number of pauses at the highest concentration (5.6 μmoles; McLaughlin et al., 2008), while ethanol and acetaldehyde did not. Moreover, acetate at the highest doses (5.6 and 8.8 μmoles) was also the most efficacious of the three substances at suppressing lever pressing in an operant schedule of reinforcement that generates very low rates of response, and thus is very difficult to suppress [i.e., the differential-reinforcement-of-low-rates-of-responding (DRL) 30 s schedule, Arizzi et al., 2003].

While in the present experiments acute administration of acetate was demonstrated to suppress locomotion, at least in rats, chronic administration of acetate in the drinking water for 15 days did not change locomotion on its own. Nevertheless, it did reduce ethanol-induced locomotion in the open field (see Figure 3). Thus, chronic pre-exposure to a low dose of acetate made animals more resistant to the stimulating effects of medium doses of ethanol in mice. Acetate, however, does not seem to mediate other ethanol well known effects, such as the anxiolytic response which acetaldehyde has been demonstrated to regulate (Correa et al., 2003, 2008; Escrig et al., 2007, 2012). Acutely administered acetate (50–200 mg/kg, IP) did not alter the behavior of mice in either the elevated plus maze or the dark and light box (Escrig et al., 2007, 2012). The same pattern of effects was observed in the interior part of an OF (Correa et al., 2003). Acutely administered acetate ICV at doses similar to the present ones (0.35–2.8 μmoles) did not modify anxiety measures in the open field in rats, although it reduced locomotion (Correa et al., 2003). Moreover, in the present results, mice exposed to acetate for 15 days did not show changes in the anxiolytic response in the dark/light box after ethanol administration. The dose of ethanol used (1.0 g/kg) has previously been demonstrated to have a potent anxiolytic effect under the present conditions (Correa et al., 2008; Escrig et al., 2012). Unfortunately, that anxiolytic effect was very mild in the present results, thus we cannot rule out this fact as the lack of interaction. In summary, although acetate has been shown to be involved in the locomotor suppressing effects of ethanol in mice (Israel et al., 1994) and rats (Correa et al., 2003; Arizzi-LaFrance et al., 2004; present results), it does not seem to mediate ethanol's anxiolytic actions (Correa et al., 2003), nor does it seem to be involved in the anxiogenic response produced by a bolus injection of acetaldehyde in the periphery (Escrig et al., 2012).

The higher levels of acetate that accumulate in the brain after repeated administration of ethanol (3.5 g/kg, IP, during 7 days) seem to mediate tolerance to LORR induced by an acute dose of ethanol (3.5 g/kg) in outbred rats (Kiselevski et al., 2003). Moreover, there is evidence that higher amounts of acetate are formed in SS rats that have an inborn tolerance to hypnotic doses of ethanol compared to the LS substrain (Zimatkin et al., 2011). In the present studies with mice, the doses achieved after consuming water with acetate concentrations of 500 and 1000 mg/l are significantly lower (around 30 and 65 mg/kg per day, respectively). Thus, the lack of effects in latency and duration of LORR after acute administration of the high doses of ethanol (4.0 and 4.5 g/kg) could be due to the fact that the doses achieved after consuming these concentrations of acetate are significantly lower than the ones used in other studies. Also, these discrepancies in results could be due to species differences; mice been shown to be more resistant than rats to the suppressive effects of ethanol and acetate. However, our results on number of animals achieving LORR (Figure 4) indicate that chronic acetate provides some sort of resistance in mice to the hypnotic effects of ethanol.

**Figure 7:**
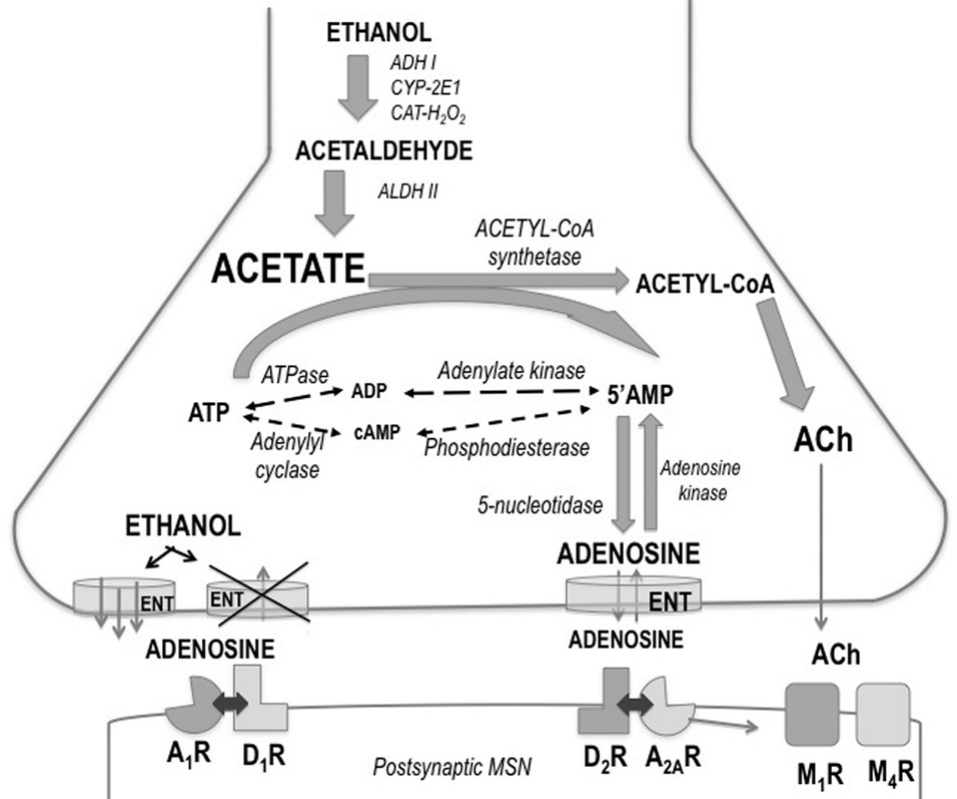
**Schematic drawing showing ethanol regulation of adenosine production, release, and uptake in striatum**. Abbreviations: A_1_R and A_2A_R, adenosine receptors; ACh, acetylcholine; ADH, alcohol dehydrogenase; ALDH, aldehyde dehydrogenase; ATP, adenosine triphosphate; AMP, adenosine monophosphate; CAT-H_2_O_2_, catalase; CYP-2E1, cytochrome P4502E1; D_1_R and D_2_R, dopamine receptors; ENT, equilibrative nucleoside transporters; M_1_R and M_4_R, muscarinic receptors; MSN, medium spiny neuron.

The precise brain areas and neural mechanisms through which acetate produces its potent suppression of motor activity are not known. A potential neuroanatomical locus for the locomotor actions of acetate, ethanol and acetaldehyde was previously found (Arizzi-LaFrance et al., 2004, 2006). Acetate injected into the substantia nigra pars reticulata of the mesencephalon produced a slight locomotor suppression (Arizzi-LaFrance et al., 2004) in contrast to the clear stimulation demonstrated for ethanol and acetaldehyde (Arizzi-LaFrance et al., 2006). Concentrations of several neurotransmitters such as acetylcholine (ACh) and adenosine seem to be modulated by the production of acetate. These hypothetical mechanisms are summarized in Figure 7. Acetate has demonstrated to increase the formation of adenosine (Dar et al., 1983; Phillis et al., 1992; Carmichael et al., 1993; Israel et al., 1994; Kiselevski et al., 2003). Ethanol increases adenosine levels by acting as a precursor through the production of acetate (Orrego et al., 1988; Carmichael et al., 1991). High doses of sub-chronically administered ethanol have been demonstrated to increase acetate, adenosine, and ACh, as well as several other biochemical factors responsible of acetate, in several areas of the brain (Kiselevski et al., 2003). It has also been suggested that ethanol as well as acetate can block adenosine uptake into the neuron (Fredholm and Wallman-Johansson, 1996; Kiselevski et al., 2003; Correa and Font, 2008), thus increasing extra-synaptic adenosine levels. Adenosine has been implicated in multiple behaviors including sleep, arousal, and motor activity (Huston et al., 1996; Iversen et al., 2009). There is evidence that adenosine may contribute to some behavioral effects of ethanol such as sedation, and motor suppression or incoordination (Proctor et al., 1985; Clark and Dar, 1988, 1989; Dar, 1990, 1993, 2000; Carmichael et al., 1991; Meng and Dar, 1995; Campisi et al., 1997; Barwick and Dar, 1998). Motor incoordination induced by ethanol is controlled by adenosine in the striatum and cerebellum (Dar, 1993; Meng and Dar, 1995). Studies also indicate that adenosine receptor activation provides a major contribution to motor suppressant effects of low concentrations of ethanol when the production of acetate is near maximal (Carmichael et al., 1993; Israel et al., 1994). At higher doses of ethanol, such as the ones used in LORR studies, the role of the acetate–adenosine system is proportionately reduced (Israel et al., 1994). As the acetate level increases after high doses of ethanol, the activation of acetyl–CoA synthetase would be expected and the formation of ACh is then potentiated (Kiselevski et al., 2003). Acetate induced increases in ACh in cerebral cortex have been associated to tolerance to ethanol-induced LORR (Zimatkin et al., 2011). Thus, the present results suggest that an increase in ACh/adenosine content may be responsible for the effects of acetate on locomotor suppression, and for blocking the stimulation of locomotion induced by ethanol and increasing resistance to achieve LORR. Further studies about the involvement of ACh, adenosine, and their subtype-receptors in these actions of acetate are warranted.

The relevance of the present acetate results (i.e., suppression of locomotion, blockade of ethanol stimulation) is related to the suggestion that two pharmacological effects that may be particularly relevant for alcohol consumption are behavioral stimulation and sedation (King et al., 2002, 2011). In general, doses of ethanol that produce more stimulation are more likely to be consumed. Subjects report that their typical drinking bout is in the dose range that was considered as having activating or disinhibiting effects (King et al., 2002, 2011). Sedative or suppressing effects on activation may also influence drinking behavior; anticipated sedative effects vary inversely with alcohol consumption (Earleywine and Martin, 1993) and heavier drinkers anticipate fewer sedative effects of alcohol than lighter drinkers (O'Malley and Maisto, 1984). Thus, sedative effects seem to prevent self-administration of ethanol, and stimulant effects can foster consumption of this drug. In agreement with these hypotheses, rats do not self-administer acetate ICV under the same conditions that lead to ethanol or acetaldehyde self-administration (Rodd-Henricks et al., 2002), and acetate does not stimulate locomotion under the same conditions that ethanol and acetaldehyde do (Correa et al., 2003).

### Conflict of interest statement

The authors declare that the research was conducted in the absence of any commercial or financial relationships that could be construed as a potential conflict of interest.
